# Vulnerability factors for mephedrone-induced conditioned place preference in rats—the impact of sex differences, social-conditioning and stress

**DOI:** 10.1007/s00213-021-05910-y

**Published:** 2021-07-15

**Authors:** Olga Wronikowska, Maria Zykubek, Łukasz Kurach, Agnieszka Michalak, Anna Boguszewska-Czubara, Barbara Budzyńska

**Affiliations:** 1 Chair and Department of Medical Chemistry, Medical University of Lublin, Chodzki 4a street, 20-093 Lublin, Poland; 2 Independent Laboratory of Behavioral Studies, Medical University of Lublin, Chodzki 4a street, 20-093 Lublin, Poland

**Keywords:** Mephedrone, Reward, Sex, Social interactions, Stress, Corticosterone

## Abstract

**Rationale:**

Mephedrone is a frequently overused drug of abuse that belongs to the group of novel psychoactive substances. Although its mechanism of action, as well as toxic and psychoactive effects, has been widely studied, the role of different factors that could contribute to the increased vulnerability to mephedrone abuse is still poorly understood.

**Objectives:**

The aim of the presented study was to assess the impact of several factors (sex differences, social-conditioning, and chronic mild unpredictable stress — CMUS) on the liability to mephedrone-induced reward in Wistar rats.

**Methods:**

The rewarding effects of mephedrone in male and female rats were assessed using the conditioned place preference (CPP) procedure. Furthermore, the impact of social factor and stress was evaluated in male rats using social-CPP and CMUS-dependent CPP, respectively.

**Results:**

Mephedrone induced classic-CPP in female (10 mg/kg), as well as in male (10 and 20 mg/kg) rats. However, the impact of mephedrone treatment during social-CPP was highly dose-dependent as the rewarding effects of low dose of mephedrone (5 mg/kg; non-active in classic-CPP) were potentiated when administered during social-conditioning. Interestingly, social-conditioning with a higher dose of 20 mg/kg (that induced classic-CPP) was able to reverse these effects. Finally, CMUS potentiated rewarding effects of a low dose of mephedrone (5 mg/kg) and increased the level of corticosterone in rats’ prefrontal cortex and hippocampus.

**Conclusions:**

Altogether, the presented results give new insight into possible factors underlying the vulnerability to mephedrone abuse and can serve as a basis for further studies assessing mechanisms underlying observed effects.

**Supplementary Information:**

The online version contains supplementary material available at 10.1007/s00213-021-05910-y.

## Introduction

Mephedrone (4-methylmethcathinone) is a synthetic derivative of cathinone, the main alkaloid compound naturally occurring in a plant *Catha edulis.* Although mephedrone was synthesized in 1929, the European Monitoring Centre for Drugs and Drug Addiction (EMCDDA) reported its first appearance on the European drug market in 2008 (EMCDDA 2010). Since then, mephedrone consumption in Europe and the USA has been increasing systematically and developed into a serious health hazard. Mephedrone belongs to the group of synthetic cathinones, which, according to the newest EMCDDA report, is the second largest group of the overused novel psychoactive substances (NPS) reported to the EU Early Warning System (EU-EWS) (EMCDDA 2019). Mephedrone exerts its effects by alternating the level of certain monoamines in the central nervous system (CNS). It has been shown to increase serotonin (5-HT) and dopamine (DA) levels in the nucleus accumbens (NAC) (Golembiowska et al. 2016; Kehr et al. 2011), striatum, and frontal cortex in rats (Golembiowska et al. 2016). Furthermore, it has been proven to inhibit the uptake and therefore to increase the level of 5-HT, DA, and noradrenaline (NA) in the CNS by interacting with the plasma membrane monoamine transporters (Baumann et al. 2012; Hadlock et al. 2011; López-Arnau et al. 2012; Martinez-Clemente et al. 2012; Pifl et al. 2015; Shortall et al. 2013). Although mephedrone mechanism of action, as well as its toxic and psychoactive effects, have been widely studied (see Papaseit et al. 2017, for review), the role of different factors (e.g. sex, social-conditioning and stress) that could contribute to the increased vulnerability to mephedrone abuse is still poorly understood.

The assessment of the role of sex as a biological variable in preclinical studies is more and more significant (Dalla et al. 2010). In preclinical studies on addiction to psychoactive substances, there is a particular need to verify this effect in both sexes. Unfortunately, female animals are often excluded from research, which leaves large gaps in our knowledge of addiction development, treatment, and neurobiological mechanisms. Studies show that sex is one of the factors that could influence the liability to drug abuse (Festa et al. 2004) and that the pattern of drug seeking and taking is expressed differently in males and females, showing that females are more vulnerable to drug overuse (see Anker and Carroll 2011; Bobzean et al. 2014, for reviews). Data from animal studies suggest analogous sex-dependent drug-seeking and -taking behaviours. For example, it has been shown that self-administration of cocaine (Roth and Carroll 2004b; Smith et al. 2011), as well as methamphetamine (Reichel et al. 2012; Roth and Carroll 2004a), is more pronounced in female rats. Taking into account that the onset and progression of drug abuse remain divergent in both sexes, identifying biological factors underlying these discrepancies could contribute to a better understanding of sex-dependent vulnerability to drug addiction. In the presented study, we aimed to assess the rewarding effects of mephedrone in the conditioned place preference (CPP) paradigm in male and female rats in order to investigate whether animals’ response to mephedrone administration differs in both sexes.

Social context during drug taking is another crucial factor that can influence the power of rewarding experience derived from the administration of different drugs of abuse. For instance, low dose (that does not induce a classic-CPP) of cocaine (Thiel et al. 2008), as well as nicotine (Thiel et al. 2009), combined with a possibility of conspecific interaction during conditioning was able to produce a rewarding effect in the CPP paradigm in rats. Other studies have shown that social influences (housing with saline- or morphine-treated partners) can affect morphine sensitization (Hofford et al. 2012) and morphine-induced CPP in mice (Cole et al. 2013). Furthermore, social stress is capable of reinstating morphine-induced CPP (Ribeiro do Couto et al. 2006), which proves that social factors play an important role in the development of drug addiction. Based on the similarities in the chemical structure, mechanism of action, and effects on the CNS, mephedrone is frequently compared to a classic empathogenic drug, MDMA (Mead and Parrott 2020). MDMA was shown to increase prosocial behaviour in animals (Ando et al. 2006; Morley et al. 2005; Procópio-Souza et al. 2011; Ramos et al. 2013; Ramos et al. 2015; Thompson et al. 2007), as well as in humans, mainly due to the changes in the levels of oxytocin (OT), vasopressin (AVP), and 5-HT (see Kamilar-Britt and Bedi 2015, for review). Taking into account the abovementioned similarities and the fact that MDMA is able to induce social, but not classic, CPP (Ramos et al. 2015), we decided to evaluate the impact of social conditioning on the acquisition of mephedrone-induced CPP.

Another aspect that can influence the predisposition to drug abuse is a stressful experience. Studies suggest that early and adult stressful events can be perceived as risk factors for the development of drug addiction (Cadet 2016; Enoch 2011). Stress alters psychological homeostasis, which can be defined as a self-regulating process aiming to maintain stability within the physiological systems by multi-system coordination. It implies that every deviation from an established standard set point is automatically corrected to maintain a state of homeostasis (Koob and Le Moal 2001; McEwen 2000). However, while discussing the relationship between stress and substance abuse, a more complex mechanism of allostasis can be proposed. Allostasis aims to maintain stability outside of the normal homeostatic range by establishing a new set point. In the case of drug abuse, allostasis can be described as a process of maintaining reward function stability by adaptive changes in the brain reward system. It implies that in the allostatic state several different factors (such as dysregulation of reward circuits and activation of brain and hormonal stress responses) contribute to the development of chronic deviation of reward set point. In light of this definition, compulsive drug taking that results in the development of addiction is a manifestation of an allostatic state (Koob and Le Moal 2001; Roberts et al. 2000).

As the relationship between stress and mephedrone-induced behavioural response in animal models has not been studied, the main goal of the third experiment presented in this paper was to assess whether stress can potentiate rewarding effects of mephedrone. Therefore, we evaluated the impact of chronic mild unpredictable stress (CMUS) on the acquisition of mephedrone-induced CPP and assessed whether mephedrone-conditioning (at the dose that failed to produce classic-CPP) is able to induce CPP when combined with stress protocol. Furthermore, to make sure that the applied CMUS protocol indeed affected stress response, we evaluated the corticosterone level in rats’ prefrontal cortex (PFC) and hippocampus, using the enzyme-linked immunosorbent assay (ELISA).

Taken together, the presented study was undertaken to comprehensively assess the impact of several factors (sex differences, social-conditioning, and CMUS) on the vulnerability to the acquisition of mephedrone-induced CPP in rats. To achieve this goal and to characterize the pharmacological profile of mephedrone in detail, three behavioural experiments were undertaken. The revealed results give a new insight into possible factors contributing to the liability to mephedrone abuse. From the translational point of view, further evaluation of mechanisms underlying observed effects would be valuable for understanding the foundation of vulnerability to drug dependence induced by the specific companionship, environment, or experience.

## Materials and methods

### Animals

All experiments were carried out on drug-naive (8 weeks old) Wistar rats obtained from the Center for Experimental Medicine of the Medical University of Lublin. In experiment 1 (*impact of mephedrone on the sex-dependent acquisition of CPP in rats*), female rats (weighing 180–220 g; *n* = 8 per group) and male rats (weighing 200–250 g; *n* = 9 per group) were used. In experiment 2 (*impact of social conditioning on the acquisition of mephedrone-induced social-CPP),* male rats weighing 200–250 g were used (in classic-CPP: *n* = 9 per group and in social-CPP: *n* = 10 rats per group for control, mephedrone 5, 10, and 20 mg/kg and *n* = 8 for mephedrone 30 mg/kg). In experiment 3 (*impact of CMUS on the acquisition of mephedrone-induced CPP),* male rats weighing 200–250 g (*n* = 8 per group) were used. The number of animals per group was chosen in a way that enables an appropriate statistical analysis. The animals were kept under standard laboratory conditions (12-h light/dark cycle, lights on 8 a.m.; room temperature 21 ± 1 °C, the relative humidity was 50 ± 5%.) with free access to tap water and a laboratory chow (Agropol, Poland). An exception of keeping animals under standard laboratory conditions was made for the animals subjected to CMUS protocol (described in details in the “Experimental protocol and treatment” section). Animals were housed in pairs with weight- and sex-matched conspecific receiving the same treatment. All experiments were carried out between 8.30 a.m. and 4.30 p.m.

### Ethics statement

All experiments were conducted according to the National Institute of Health Guidelines for the Care and Use of Laboratory Animals and to the European Community Council Directive for the Care and Use of Laboratory Animals of 22 September 2010 (2010/63/EU). All protocols were approved by the local ethics committee: experiment 1 (permit number 53/2018; 31/2019), experiment 2 (permit number 37/2019), and experiment 3 (permit number 42/2019).

### Drugs

Mephedrone hydrochloride (Toronto Research Chemicals Inc., Canada) was administered in intraperitoneal injections (i.p.) at a volume of 2 ml/kg. Control groups received saline solutions (0.9% NaCl) via the same route of administration and at the same volume. The solutions were freshly made each day of the experiments. In experiment 1, mephedrone was administered at the doses of 5, 10, and 20 mg/kg (female rats) and at the doses of 5, 10, 20, and 30 mg/kg (male rats). In experiment 2, mephedrone was administered at the doses of 5, 10, 20, or 30 mg/kg and in experiment 3 at the dose of 5 mg/kg. In all 3 experiments, saline and mephedrone were administered for 6 consecutive days of conditioning. Drug doses were chosen based on our previous experiments and preliminary studies.

### Experimental protocol and treatment

#### CPP apparatus and software

In all 3 experiments, CPP procedure was conducted using Ugo Basile CPP system and VideoMot software. Single UgoBasile apparatus (external dimensions: 63 × 32x35cm) consists of two compartments (internal dimensions 30 × 30 × 30 cm) differing by tactile and visual stimulation (floor structures and wall patterns) which are divided by the guillotine doors. One compartment has black and white striped walls and floor with round 2-mm holes and the other one has black walls and a floor with square 10 × 10 mm holes. VideoMot software enables live tracking of the animal by 3 point detection (head/centre/tail) by a contrast filter and enables to measure time spent in each compartment, as well as the distance travelled.

#### CPP procedure

In all 3 experiments, CPP was conducted in an unbiased design where animals do not show any initial preference to either of the compartments. CPP consisted of 3 phases: pre-conditioning test, conditioning, and post-conditioning test***.*** One day prior to these phases, animals were habituated for 15 min to the apparatus to minimize the stress which could affect their behavioural response. The CPP procedure was already validated in our laboratory and conducted as previously described in detail (Biala et al. 2018) with a small modification. In all three experiments, conditioning sessions were conducted twice a day and were separated by a 4-h interval. Morning sessions took place between 8.30 a.m. and 12.30 p.m. and afternoon sessions took place between 12.30 p.m. and 4.30 p.m.

##### Experiment 1: impact of mephedrone on the sex-dependent acquisition of CPP in rats

**Fig. 1:**
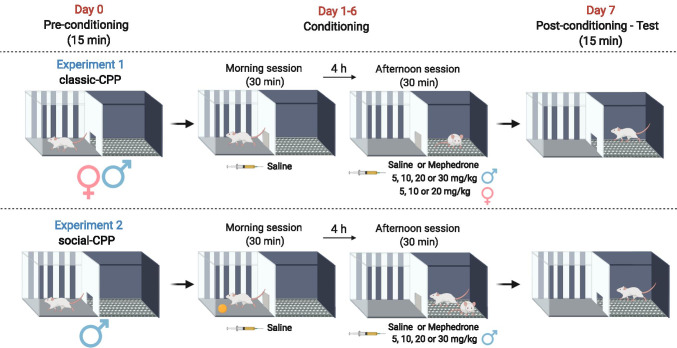
Experimental protocol of sex-dependent classic-conditioned place preference (CPP) (experiment 1) and social-CPP (experiment 2). This figure was created with BioRender.com

Since studies suggest that there might be sex-dependent differences in vulnerability to drug-induced rewarding effects, we evaluated an impact of mephedrone on the acquisition of CPP in male and female rats in an analogous manner. During the pre-conditioning phase, rats were placed individually in the apparatus with guillotine doors opened and free access to both compartments. The time spent in each compartment (initial preference) was recorded for 15 min. The next phase (conditioning) lasted 6 days and consisted of 2 sessions (morning and afternoon, each lasting 30 min). During this phase, the guillotine door was closed. In the morning session, each animal was confined in one compartment and received a saline injection, whereas in the afternoon session each rat was confined in the other compartment and received saline (control) or mephedrone injection. Female rats received mephedrone at the dose of 5, 10, or 20 mg/kg and male animals received mephedrone at the doses of 5, 10, 20, or 30 mg/kg. Within each group, animals were randomly divided into 2 groups conditioned in different compartments. In the initial design, all four doses (5, 10, 20, and 30 mg/kg) were planned to be administered to both sexes. Nonetheless, female rats that were administered with a dose of 20 mg/kg displayed very aggressive behaviour towards the experimenter and towards the technician staff. Thus, after consideration, we decided not to administer a higher dose of 30 mg/kg to female animals. On the 7th day, animals did not receive any injections and were subjected to the post-conditioning test. Guillotine door was opened and the time spent in each compartment was recorded for 15 min. The experimental protocol of sex-dependent CPP has been presented in Fig. 1.

##### Experiment 2: impact of social conditioning on the acquisition of mephedrone-induced social-CPP

To assess the impact of the possibilities of social interactions during conditioning on rewarding properties of mephedrone, we used the protocol of social-CPP (adapted from Ramos et al. 2015 with small modifications). In both classic- and social-CPP, pre-conditioning and post-conditioning phases are conducted in the same way; however, the main difference concerned the conditioning phase. In the applied social-CPP protocol, during the morning session animals received vehicle injection and they were confined individually to one compartment with free access to a non-social object (a classic tennis ball). During the afternoon session, animals were injected with mephedrone (5, 10, 20, or 30 mg/kg) and placed in the other compartment with a familiar, identically treated, sex- and weight-match conspecific. The partner rat remained the same for each conditioning session during the conditioning phase. The experimental protocol of mephedrone social-CPP has been shown in Fig. 1.

##### Experiment 3: impact of CMUS on the acquisition of mephedrone-induced CPP

**Fig. 2:**
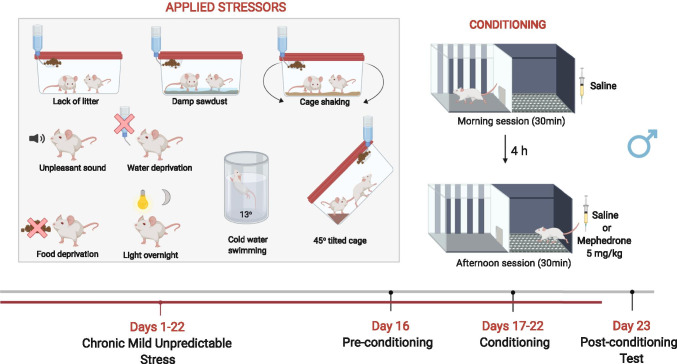
Experimental protocol of chronic mild unpredictable stress (CMUS) procedure combined with conditioned place preference (CPP) (experiment 3). This figure was created with BioRender.com

Considering the fact that stress can strongly influence humans’ as well as rodents’ behaviour, the aim of this experiment was to evaluate whether CMUS affects rats’ vulnerability to the acquisition of mephedrone-induced CPP. For this purpose, we assessed whether mephedrone at the dose that previously failed to induce classic-CPP (5 mg/kg) is able to induce CPP when combined with CMUS protocol. CMUS protocol has been designed based on the experiments previously performed and validated at our university (Biala et al. 2017, 2018), as well as based on other available data (Mineur et al. 2006; Papp et al. 1996)*.* Since the aim of the experiment was to assess the impact of stress on acquisition of mephedrone-CPP, animals were exposed to CMUS for a total amount of 22 days (15 days preceding and for 7 days during the CPP paradigm). However, to avoid the effects of acute stress, animals were not exposed to any stressors during the test day. All animals have been subjected to the same sequence of stressors which were applied once a day and consisted of (1) the lack of litter for 24 h, (2) damp sawdust for 24 h, (3) swimming in cold water (13 °C for 5 min), (4) tilted cage at 45° for 4 h, (5) lights on overnight, (6) food deprivation for 24 h, (7) water deprivation for 12 h, (8) cage shaking (4 times for 2 min in 5-min interval), and (9) unpleasant sound (4 times for 5 min in 10-min interval). The CPP procedure started on the 16th day of the experiment and was performed in an analogous manner as described for experiment 1. CMUS-exposed, as well as control non-stressed animals, were conditioned with saline or mephedrone (5 mg/kg) for 6 consecutive days (days 17–22). On the 23rd day, rats did not receive any treatment and place preference was measured during the post-conditioning test for 15 min. For the sake of clarity and transparency, a detailed schedule of the applied CMUS paradigm has been added to Supplementary Materials. The experimental protocol of CMUS procedure combined with CPP has been presented in Fig. 2.

#### Evaluation of a locomotor activity

As the determination of locomotor activity is crucial for the paradigms when mobility can influence observed results, we have also evaluated this parameter. To determine the impact of studied factors on locomotor activity of saline- or mephedrone-conditioned rats, locomotion was recorded using an Ugo Basile CPP system and VideoMot software. Total horizontal activity (distance travelled in metres) was recorded for 15 min on the last day (test day) of each experiment (24 h after last mephedrone administration).

#### Determination of corticosterone level in brain tissue homogenates

In order to confirm that the implemented CMUS protocol certainly exerted a stress effect, we evaluated corticosterone level in brain tissue homogenates in saline- and mephedrone-conditioned animals subjected, or not subjected, to CMUS, with ELISA method. After 22 days of stress protocol (on the 23rd day of experiment 3), animals were decapitated immediately after the post-conditioning test and the hippocampus and PFC were carefully dissected and washed in ice-cold saline (*n* = 8 samples per group). Samples, stored at − 80 °C, were homogenized in enzyme immunoassay buffers of the kit (Cayman, MI, USA) and centrifuged at 1500 rpm for 15 min at + 4 °C. Supernatants were treated according to the manufacturer’s protocol.

### Statistical analysis

For the CPP paradigm, data were analysed by one-way or two-way analysis of variance (ANOVA) followed by the adjustments for multiple comparisons. The results are expressed as means + standard error of the mean (SEM) of scores (i.e., the differences between post-conditioning and pre-conditioning time spent in the drug-associated compartment). For the evaluation of locomotor activity, the data were analysed using one- or two-way ANOVA (with the adjustments for multiple comparisons) or t-test Student, when appropriate. The results are expressed as means + SEM of distance travelled measured in metres for 15 min. For the ELISA determination of corticosterone level in brain tissue homogenates, data were analysed using two-way ANOVA with multiple comparisons and the results are expressed as picogrammes of corticosterone per milligramme of tissue. In all experiments, post hoc comparison of means was carried out with Tukey’s test. All statistical tests were performed using GraphPad Prism version 8.0.1 for Windows (GraphPad Software, USA). The confidence limit of P < 0.05 was considered statistically significant.

## Results

### Mephedrone-induced CPP in male and female rats

**Fig. 3:**
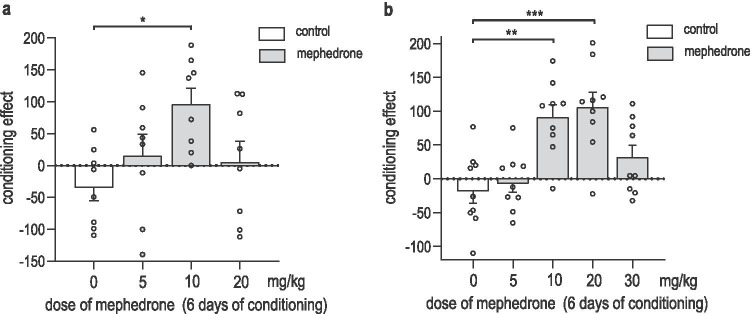
Mephedrone-induced conditioned place preference (CPP) in female (**a**) and male (**b**) rats. Animals were subjected to pre-conditioning (day 0), 6 days of mephedrone-conditioning (5–30 mg/kg) (days 1–6), and post-conditioning test (day 7). Data represent means + SEM and are expressed as the difference (in seconds) between post-conditioning and pre-conditioning time spent in the drug-associated compartment. *n* = 8 female and *n* = 9 male rats per group; *P < 0.05, **P < 0.01, ***P < 0.001 (Tukey’s test)

Figure 3 shows the effect of mephedrone-conditioning in female (Fig. 3a) and male (Fig. 3b) rats. One-way ANOVA showed a significant effect of mephedrone treatment in female rats: [F (3, 28) = 3.528; *P* = 0.0267]. Mephedrone administration at the dose of 10 mg/kg during 6 days of conditioning induced a clear place preference in female rats, as post hoc analyses showed significant differences in scores between saline-conditioned and mephedrone-conditioned group (*P* = 0.0185, post hoc Tukey’s test). Mephedrone at the doses of 5 and 20 mg/kg did not induce CPP in female rats. One-way ANOVA showed significant treatment effect also in male rats: [F (4, 40) = 9.000; *P* < 0.0001]. Mephedrone-conditioning at the doses of 10 and 20 mg/kg induced CPP in male rats (*P* = 0.0016; *P* = 0.0003, respectively; post hoc Tukey’s test); however, we did not observe place preference triggered by mephedrone-conditioning at doses of 5 and 30 mg/kg.

### Effects of social conditioning on mephedrone-induced social-CPP in male rats

**Fig. 4:**
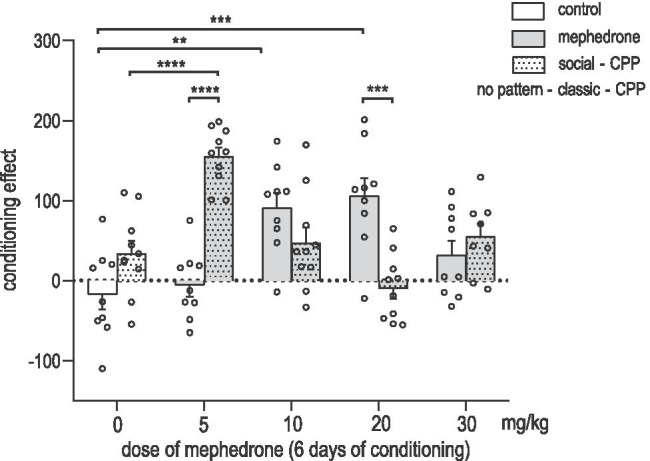
Mephedrone effects on classic- and social-conditioned place preference (CPP) in male rats. Data represent means + SEM and are expressed as the difference (in seconds) between post-conditioning and pre-conditioning time spent in the drug-associated compartment. Classic-CPP: *n* = 9 rats per group; Social-CPP: *n* = 10 rats per group (for control, mephedrone 5, 10, and 20 mg/kg) and *n* = 8 (for mephedrone 30 mg/kg); **P < 0.01; ***P < 0.001; ****P < 0.0001 (Tukey’s test)

Figure 4 indicates the effects of social conditioning on the acquisition of mephedrone-induced social-CPP in rats [two-way ANOVA: interactions treatment-social-conditioning: F (4, 83) = 18.85; P < 0.0001; treatment: F (4, 83) = 4.823; *P* = 0.0015; social-conditioning: F (1, 83) = 1.919; *P* = 0.1697]. We confirmed that mephedrone (10 and 20 mg/kg) induced classic-CPP when compared with saline-conditioned rats (*P* = 0.00121; *P* = 0.0001, respectively; post hoc Tukey’s test). Mephedrone at the dose of 5 and 30 mg/kg did not induce CPP. Furthermore, the possibility of social interactions during conditioning dose-dependently reversed the score values obtained in social-CPP showing that social-conditioning with mephedrone at the dose of 5 mg/kg was able to induce CPP when compared to saline-conditioned rats in social-CPP (P < 0.0001; post hoc Tukey’s test) and when compared to mephedrone-conditioned (5 mg/kg) rats in classic-CPP (P < 0.0001; post hoc Tukey’s test). Interestingly, social conditioning with mephedrone at the dose of 20 mg/kg reversed the rewarding effect observed in classic-CPP and did not produce a rewarding effect when compared with mephedrone classic-conditioned rats at the dose of 20 mg/kg (*P* = 0.0003; post hoc Tukey’s test).

### Effects of CMUS on mephedrone-induced CPP in male rats

**Fig. 5:**
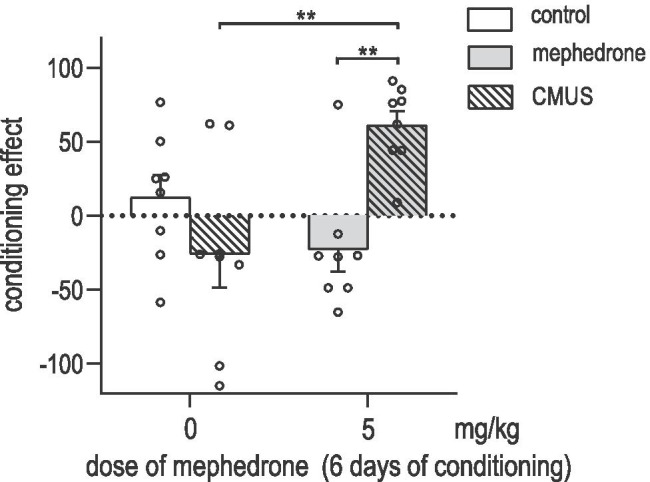
Influence of chronic mild unpredictable stress (CMUS) on the acquisition of mephedrone-induced conditioned place preference (CPP) in male rats. Animals were subjected to CMUS protocol for 22 days (days 1–22) and to pre-conditioning (day 16), 6 days of mephedrone-conditioning (5 mg/kg) (days 17–22) and post-conditioning test (day 23). Data represent means + SEM and are expressed as the difference (in seconds) between post-conditioning and pre-conditioning time spent in the drug-associated compartment. *n* = 8 rats per group; **P < 0.01 (Tukey’s test)

Figure 5 indicates the effects of CMUS on mephedrone-induced CPP in rats, [two-way ANOVA: interaction treatment-CMUS: F (1, 28) = 13.81; *P* = 0.0009; treatment: F (1, 28) = 2.481; *P* = 0.1265; CMUS: F (1, 28) = 1.952; *P* = 0.1733]. Firstly, we confirmed previously obtained results that mephedrone administered at a dose of 5 mg/kg does not induce CPP. Secondly, we showed that CMUS significantly affected the effects observed in CPP as CMUS-exposed rats conditioned with mephedrone at the dose of 5 mg/kg showed a statistically significant increase in CPP score values when compared to CMUS-exposed saline-conditioned animals and when compared to non-stressed mephedrone-conditioned (5 mg/kg) animals (*P* = 0.0043; *P* = 0.006, respectively; post hoc Tukey’s test).

### Evaluation of a locomotor activity

Table 1 indicates the effect of mephedrone-conditioning on male and female rats mobility measured during post-conditioning test of experiment 1 (24 h after last mephedrone administration). One-way ANOVA did not reveal any statistically significant differences in locomotor activity neither in female [F (3, 28) = 2.129; *P* = 0.1190] nor in male [F (4, 40) = 2.313; *P* = 0.0742] animals, when compared to saline-conditioned female or male animals, respectively. Nevertheless, t-test showed significantly higher mobility in the control group of male rats (*P* = 0.0018) when compared to the control group of female rats [unpaired T-test Student: saline female vs male: t = 3.782, df = 15; *P* = 0.0018]. Locomotor activity in male and female rats conditioned with mephedrone at all of the tested doses did now differ between sexes [unpaired t-test Student: mephedrone 5 female vs male: t = 2.019, df = 15; *P* = 0.0618; mephedrone 10 female vs male: t = 2.106, df = 15; *P* = 0.0525; mephedrone 20 female vs male: t = 1.718, df = 15; *P* = 0.1063]. In experiment 3, neither mephedrone nor CMUS affected animals mobility in CMUS-dependent CPP [two-way ANOVA: interaction treatment-CMUS: F (1, 28) = 1.063; *P* = 0.3113; treatment: F (1, 28) = 3.202; *P* = 0.0844; CMUS: F (1, 28) = 3.258; *P* = 0.0818] when measured on day 23 of CMUS protocol, 24 h after last mephedrone administration (Table 2).

**Table 1:** Effect of 6 days of mephedrone administration on locomotor activity in female and male rats, recorded as distance travelled (in metres) during 15 min on the test day (7th) of conditioned place preference (CPP) (24 h after last mephedrone administration). Results are expressed as mean + SEM (*n* = 8 female rats and *n* = 9 male rats per group). No significant changes in locomotor activity were observed in either of the analysed groups within the same sex when compared to saline-conditioned rats (one-way ANOVA). Statistically significant increase in locomotor activity was observed in male rats when compared to female rats in the control group (**P < 0.01; t-test Student)

Distance travelled (m) day 7					
Sex	Saline	Mephedrone 5	Mephedrone 10	Mephedrone 20	Mephedrone 30
Females	7.64 ± 0.46	8.24 ± 0.9	8.78 ± 0.17	8.19 ± 0.26	Not applicable
Males	9.4 ± 0.17**	8.96 ± 0.18	9.38 ± 0.22	8.78 ± 0.17	8.19 ± 0.26

**Table 2:** Effect of chronic mild unpredictable stress (CMUS) on locomotor activity in saline- and mephedrone-conditioned rats recorded as distance travelled (in metres) during 15 min, on the test day (23rd), 24 h after last mephedrone administration. Results are expressed as mean + SEM; *n* = 8 rats per group. No significant changes in locomotor activity were observed for either of the analysed groups (two-way ANOVA)

Distance travelled (m) day 23		
Conditioning days 17–22	Saline	Mephedrone 5
No CMUS	9.33 ± 0.17	8.64 ± 0.23
CMUS	9.52 ± 0.3	9.33 ± 0.26

### Determination of corticosterone level in brain tissue homogenates

**Fig. 6:**
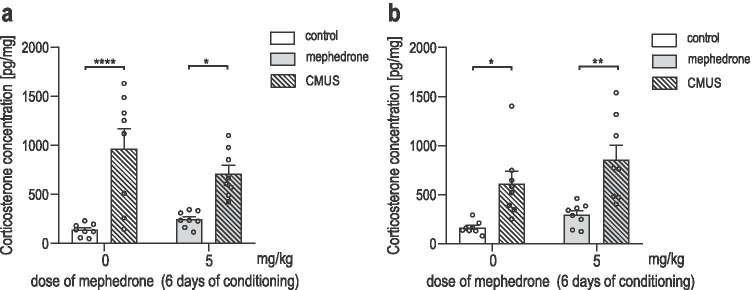
Corticosterone concentration in the prefrontal cortex (**a**) and the hippocampus (**b**) of male rats subjected to 22 days of chronic mild unpredictable stress (CMUS) and 6 days of saline- or mephedrone-conditioning (5 mg/kg). Brain tissue homogenates were collected on the 23rd day of CMUS protocol, immediately after the post-conditioning test. Data are expressed as a means + SEM of corticosterone concentration (pg/mg of tissue); *n* = 8 samples per group; *P < 0.05; **P < 0.01; ****P < 0.0001 (Tukey’s test)

Figure 6 shows the effect of CMUS and mephedrone administration (5 mg/kg) on corticosterone concentration in the (a) PFC [two-way ANOVA: interaction treatment-CMUS: F (1, 28) = 2.631; *P* = 0.1160; treatment: F (1, 28) = 0.4243; *P* = 0.5201; CMUS: F (1, 28) = 33.45; *P* < 0.0001] and (b) hippocampus [two-way ANOVA: interaction treatment-CMUS: F (1, 28) = 0.3289; *P* = 0.5709; treatment: F (1, 28) = 3.453; *P* = 0.0737; CMUS: F (1, 28) = 25.12; *P* < 0.0001]. Post hoc Tukey’s test revealed that the concentration of corticosterone in the PFC is significantly elevated in both saline- and mephedrone-conditioned CMUS-exposed rats when compared to non-stressed saline-conditioned (P < 0.0001) and non-stressed mephedrone-conditioned (*P* = 0.0310) rats, respectively. Similarly, post hoc Tukey’s test revealed that the concentration of corticosterone in the hippocampus is also significantly elevated in both: saline- and mephedrone-conditioned CMUS-exposed rats when compared to non-stressed saline-conditioned (*P* = 0.0196) and non-stressed mephedrone-conditioned (*P* = 0.0026) rats. Corticosterone level in rats not subjected to CMUS did not differ between saline- and mephedrone-conditioned rats in either of tested structures.

## Discussion

The abuse of NPS is becoming a significant burden among young people, leading to changes in their personalities manifested by destructive and aggressive behaviours. The effects of NPS abuse have not been sufficiently investigated, especially in the context of understanding the sex-dependent differences, the social aspect of the reward mechanism, as well as the influence of stress on drug abuse. Thus, to the best of our knowledge, we have undertaken such a study for the first time. Our research hypotheses were based on previously reported sex-dependent differences in the liability to drug abuse, the similarity of the chemical structure, mechanism of action, and the effects on CNS of mephedrone and MDMA (Mead and Parrott 2020) and on our earlier research assessing stress impact on the rewarding effects of nicotine (Biala et al. 2018). Altogether, the presented research gives a new insight into possible factors contributing to the liability to mephedrone abuse and can serve as a basis for further studies assessing mechanisms underlying the observed effects.

### Impact of mephedrone on the sex-dependent acquisition of CPP in rats

It has been previously shown that the rewarding effects of drugs can be sex-dependent and expressed differently in male and female animals (Reichel et al. 2012; Roth and Carroll 2004a, 2004b; Smith et al. 2012). The reinforcing properties of mephedrone in male animals have been widely studied in intracranial self-stimulation test (Bonano et al. 2014; Nguyen et al. 2016; Robinson et al. 2012; Suyama et al. 2019), as well as in self-administration paradigm (Marusich et al. 2019a). Here, we confirmed priorly reported (Ciudad-Roberts et al. 2015; Karlsson et al. 2014; Lisek et al. 2012) mephedrone rewarding properties in CPP in male rats, showing that mephedrone at the doses of 10 and 20 mg/kg (but not 5 and 30 mg/kg) induced CPP. In female rodents, mephedrone has been shown to induce drug reward so far only in self-administration paradigm (Creehan et al. 2015; Marusich et al. 2019b). In the presented research, we confirmed these properties also in the CPP test, showing that mephedrone produced CPP at the dose of 10 mg/kg (but not 5 and 20 mg/kg).

Regarding the fact that animals’ mobility can affect results observed in CPP, locomotor activity cannot be omitted while discussing the CPP outcomes. The possibility of simultaneous measurement of CPP values and locomotor activity is an undeniable advantage of the applied protocol as it did not expose animals to more stress factors connected with the alternative of conducting two separate tests. The effect of mephedrone administration on rodents mobility has been previously reported in male rodents, showing an increase in locomotor activity when tested immediately after mephedrone administration in acute (Ciudad-Roberts et al. 2015; López-Arnau et al. 2012; Martínez-Clemente et al. 2013; Miller et al. 2013; Šíchová et al. 2018), as well as repeated (Angoa-Pérez et al. 2012; Mayer et al. 2016; Shortall et al. 2016) treatment regimen. Moreover, it has been reported that mephedrone-induced hyperlocomotion lasted approximately 40–120 min, depending on administered dose (Ciudad-Roberts et al. 2015; Gatch et al. 2013; Martínez-Clemente et al. 2013). In our study, locomotor activity was assessed for 15 min during the test day, 24 h after the last mephedrone administration. The results revealed that mephedrone affected locomotor activity neither in female nor in male rats, when compared to saline-conditioned female or male animals, respectively. Considering the priorly reported length of mephedrone-induced increase in animal mobility, the lack of hyperlocomotion in both sexes is in line with currently available reports. Interestingly, higher mobility was observed in male animals when compared to female animals in saline-treated animals, suggesting that the animals’ mobility is sex-dependent and male rats, in general, may display increased locomotor activity than females.

Our comprehensive evaluation revealed that mephedrone exerts rewarding effects in the CPP paradigm in both sexes creating an inverted U-shaped response. The observed bell curve suggests that the administered dose plays a pivotal role in rewarding effects of mephedrone. We may propose a hypothesis that lower doses are insufficient to trigger the response, whereas the highest doses may induce stereotypy. Although the assessment of stereotyped behaviour was not performed in this study, the similar syndrome was reported following administration of high doses of other psychostimulants (Huang et al. 2012; O’Loinsigh et al. 2001). The reported mephedrone-induced CPP is not only dose- but also sex-dependent, as the most robust response was observed at different doses in male and female animals. The most pronounced effect was produced at a lower dose in female animals (10 mg/kg) than in male animals (20 mg/kg). Additionally, mephedrone at the dose that produced statistically the strongest response in male rats displayed no significant effect in female rats. This indicates that female animals could be more vulnerable to the drug effect and confirms the hypothesis that sex may be one of the factors altering the liability to mephedrone abuse. Hormonal changes in females could be at least one of the possible causes of the observed distinction; however, the relationship between mephedrone effects and ovarian hormones has not been investigated yet. So far, it has been reported that estradiol can potentiate mephedrone-induced disruption in spatial memory in ovariectomized rats (Weed et al. 2014) and that mephedrone exposure during the gestational period impaired learning and memory in mice offspring through hippocampal damage (Naseri et al. 2018). Nevertheless, previous studies of other drugs of abuse showed that ovariectomized and castrated rats show different rates of acquisition of cocaine self-administration (Hu et al. 2004), suggesting that sex hormones may not be crucial for differences in the effect of drugs in female and male rodents. Thus, an alternative reason for the observed effects may lie in the differences in the pharmacokinetics of psychostimulant drugs. The studies showed that female rats eliminate drugs at a slower rate than males, which results from the different activity of metabolizing enzymes, e.g., cytochrome P-450 (Kato 1974; Mugford and Kedderis 1998). Therefore, the effects of drugs may be greater and/or may last longer (Festa et al. 2004). We may hypothesize that the level of mephedrone can remain at a higher value in the blood and brain of female rats; thus, stereotypic behaviours may occur after administration of lower doses than in males. Nevertheless, since there is insufficient data to fully support this hypothesis, further and more detailed studies, directly comparing blood and brain levels of mephedrone in both sexes, are needed.

The animal studies are consistent with those performed in humans showing that the pattern of drug seeking and taking can be expressed differently in males and females, making sex a vulnerability factor in drug abuse (see Anker and Carroll 2011; Bobzean et al. 2014, for reviews). In clinical reports, women were reported to initiate drug use at an earlier age than men (Chen and Kandel 2002) and were more likely to transit from casual to binge-like patterns of drug taking (Lynch et al. 2002; Mann et al. 2005). Interestingly, we also observed that female rats administered with mephedrone at the dose of 20 mg/kg displayed aggressive behaviour after conditioning. Since this observation has not been expected, it was not measured and therefore, we cannot comprehensively discuss it. Nevertheless, it suggests that mephedrone can induce aggression in sex- and dose-dependent manner. Since available reports suggest that mephedrone can trigger aggression in humans (Dolengevich-Segal et al. 2016), it would be of particular importance to determine the underlying mechanisms of this behaviour in order to prevent this from happening.

### Impact of social-conditioning on the acquisition of mephedrone-induced social-CPP

Although the impact of social context and its effect on drug abuse has been frequently reported for other drugs of abuse (Cole et al. 2013; Hofford et al. 2012; Ribeiro do Couto et al. 2006; Thiel et al. 2008, 2009), the relationship between social context and rewarding effects of mephedrone is underexplored. So far, only one study has tried to evaluate the impact of acute mephedrone administration on social behaviour in the social interactions test, suggesting a possible mephedrone-induced reduction in social preference; however, the results remained statistically insignificant (Motbey et al. 2012). Nevertheless, taking into account the fact that mephedrone is frequently compared to MDMA, a classic empathogenic drug associated with an increase in prosocial behaviours in animal models, as well as in humans (see Kamilar-Britt and Bedi 2015, for review), we hypothesized that mephedrone may display a similar effect. The presented results showed that the company of identically treated conspecific during 6 days of mephedrone-conditioning had a dose-dependent impact on the acquisition of the rewarding effect of mephedrone. The biphasic response showed that the social-conditioning increased the rewarding effect as the low dose of mephedrone 5 mg/kg (that failed to induce classic-CPP) was able to induce social-CPP. Interestingly, social-conditioning with mephedrone in a higher dose of 20 mg/kg (that induced classic-CPP) significantly reduced a rewarding effect. These ambiguous results clearly suggest that the impact of social-conditioning on the rewarding effects of mephedrone is highly dose-dependent. The rewarding effects of low doses of mephedrone may be potentiated when combined with the presence of the identically treated conspecific; however, at the same time, social-conditioning with higher doses of mephedrone dose-dependently attenuates the rewarding effect observed in classic-CPP. Although mechanisms responsible for mephedrone effects modulated by different social contexts have not been investigated yet, several potential molecular targets can be suspected based on the available data on MDMA-dependent changes in social behaviour (Hunt et al. 2011; Ramos et al. 2013, 2015; Thompson et al. 2007). Particularly, the impact of OT, AVP, 5-HT, and DA on mephedrone-induced ambiguous social effects is currently under investigation. Furthermore, according to Trezza et al. (2009), one of the possible explanations of the observed dose-dependent differences may be reflected in the changes or suppression of social interaction between animals. However, since a limitation of our study is that we did not measure social behaviour during conditioning, further research is needed to support this hypothesis.

The observed biphasic response supports the assumption that social context during drug administration significantly affects the experience derived from drug seeking. Furthermore, comparing to available literature that clearly indicates that MDMA increases prosocial behaviour in animals (Ando et al. 2006; Morley et al. 2005; Procopio-Souza et al. 2011; Ramos et al. 2013; Ramos et al. 2015; Thompson et al. 2007), we may derive an assumption that although mephedrone and MDMA share many similarities (Mead and Parrott 2020), these drugs cannot be perceived as analogues in terms of their effects. The reflection of these differences is also observed in humans, as it was reported that although the acute desired effects of mephedrone are often rated to be very similar to those of MDMA (Kapitány-Fövény et al. 2013), the acute adverse effects are generally rated more negatively (Matthews et al. 2017). Furthermore, users report that the duration of action is significantly shorter with mephedrone use, attributing towards a pattern of binge use among users (see Mead and Parrott 2020 for review).

### Impact of CMUS on the acquisition of mephedrone-induced CPP and PFC/hippocampal corticosterone level

Stress is another significant factor that could affect the liability for drug abuse. Clinical studies have shown that stress can exacerbate craving for drug seeking (Blaine and Sinha 2017; Preston et al. 2018a, 2018b). The effects observed in humans are consistent with the numerous studies in animal models which have shown that stress exposure can alter the response elicited by the administration of addictive substances. For example, CMUS, as well as acute stress, was able to enhance and reinstate nicotine seeking behaviour (Biala et al. 2017; Brielmaier et al. 2012; Leão et al. 2009; Smith et al. 2012; Yamada and Bruijnzeel 2011) and magnify the rewarding effects and self-administration of ethanol (Sperling et al. 2010). Furthermore, stress was able to potentiate the rewarding effects of cocaine in both of the abovementioned paradigms (Kreibich et al. 2009; McLaughlin et al. 2006). Although there is strong evidence supporting a significant relationship between stress exposure and drug addiction, the stress-dependent consequences on mephedrone-induced rewarding effects are unknown. Thus, the last part of our studies aimed to test the hypothesis whether stress can increase the vulnerability to mephedrone-induced reward in rats.

Random and unpredictable exposure to different stressors induces a state of anhedonia and triggers behavioural changes that could influence the effects of other factors (i.e., drug seeking) (Antoniuk et al. 2019; Yan et al. 2010). Here, we aimed to evaluate the impact of CMUS on the acquisition of mephedrone-induced CPP. As the results showed, mephedrone (at the dose of 5 mg/kg that failed to produce classic-CPP) was able to induce CPP when combined with stress protocol with no significant changes in locomotor activity. The observed behavioural outcome confirms the hypothesis that stress can increase the rewarding effects of mephedrone, and therefore it can influence the liability for mephedrone overuse.

Although the behavioural results showed that exposure to CMUS facilitated the acquisition of mephedrone-induced CPP, to exhaustively confirm that the observed effects were stress-dependent, the biochemical determination of corticosterone level was performed. Corticosterone is an adrenal glucocorticosteroid and it is perceived as the main stress hormone in rodents and its level is frequently measured as an indicator of elevated stress level (Gong et al. 2015). Although corticosterone level is often analysed in plasma, it has been also reported that cocaine-induced changes in corticosterone level that matched their behavioural effects were undetectable in plasma but were found in the medial PFC (Keller et al. 2017). Additionally, as the tendency of limbic structures, the hippocampus and septum, to concentrate and retain labelled corticosterone was observed (McEwen et al. 1969), we decided to determine corticosterone concentration in the brain (specifically, in the PFC and hippocampus). These brain structures are not only sensitive to corticosterone level changes but also are involved in the development of addiction (McEwen et al. 1969; Keller et al. 2017; Piskunov et al. 2016; Volkow et al. 2019). PFC is a structure of mesocorticolimbic drug reward system that receives projections from midbrain DA neurons and projects to other key dopaminergic regions, enhancing the drug-derived reward (Chau et al. 2018). Furthermore, drug exposure can also enhance hippocampal function, leading to reinforcement of drug addictive potential. Moreover, DA neurons of the ventral tegmental area (VTA), another structure representing the brain’s drug reward system, were shown to project to the hippocampus. Therefore, this structure is proven to mediate emotional and memory responses contributing to the formation of drug-related associations and leading to the development of addiction (Volkow et al. 2019). The analysis of the corticosterone level in rats PFC and hippocampus was performed using ELISA which is a well-established immunological assay commonly used to measure the level of antibodies or antigens in biological samples. The results revealed that 22 days of CMUS significantly increased the corticosterone level in the PFC and hippocampus in both, saline- and mephedrone-conditioned rats. Nevertheless, the corticosterone level in rats not subjected to CMUS did not differ between saline- and mephedrone-conditioned rats in either of the tested structures, proving that a low dose of mephedrone (5 mg/kg) does not induce stress response by itself. Therefore, it can be stated that the obtained behavioural response in CPP was clearly dependent on the applied CMUS protocol.

### Limitations

Although the presented research delivers interesting findings and casts new light on the vulnerability factors contributing to mephedrone abuse, we identified some limitations that should be taken into consideration and improved in future studies. Firstly, it would add great value to analyse the sex-dependent effect of mephedrone in classic-CPP using two-way ANOVA. Nonetheless, based on the reasons mentioned in the “CPP procedure” section, experiment 1, we decided not to administer a dose of 30 mg/kg to female rats. As we wished to present an inverted U-shaped response in both sexes, we chose to analyse each group separately. Secondly, it would be highly informative to complement the performed sex-dependent CPP with the assessment of mephedrone blood/brain level or metabolism rate in both sexes. Therefore, we plan to incorporate it into our future experiments. Furthermore, we believe that while analysing the impact of social factor on mephedrone-induced reward, it would be advantageous to investigate the extent and character of social interactions displayed during social-conditioning. Such analysis would help to understand the behavioural difference of the observed biphasic response and has been planned for our further research.

## Conclusions

The present study was undertaken to evaluate the impact of selected factors contributing to drug addiction, i.e., sex, social-conditioning, and stress, on the behavioural response to mephedrone and its rewarding effects in the CPP paradigm. First of all, our results indicate that sex is one of the factors that could alter the liability to mephedrone abuse. Although the rewarding effects were shown for both sexes, the most pronounced effect of mephedrone-induced CPP was observed at a lower dose in female than male rats. Unfortunately, female animals are often excluded from research. The results of our study support the assumption that sex should be considered an important biological variable in the field of preclinical studies and that researchers should not overlook this issue. Furthermore, we revealed that mephedrone affects social-CPP not only in a dose-dependent manner but also exhibits an interesting biphasic reward response in socially conditioned rats. On the one hand, the rewarding effects of a low dose of mephedrone (non-active in classic-CPP) were potentiated when administered along with social-conditioning. At the same time, social-conditioning of animals subjected to a higher dose of mephedrone (active in classic-CPP) reversed primary mephedrone-induced reward. Knowing that many psychoactive substances are taken not individually but rather in groups, we may state that it would be highly valuable for further research to incorporate a social context into experiments assessing addiction in the broad sense. Finally, we also showed that stress-induced potentiation of the rewarding effects of mephedrone is correlated with an increase in corticosterone levels in the PFC and hippocampus. Altogether, our results give a new insight into possible factors contributing to the abuse liability of mephedrone. Further investigations are of great importance for answering the question of what exact molecular mechanisms are behind described effects of mephedrone.

## Supplementary Information

Below is the link to the electronic supplementary material.

Supplementary file1 (DOCX 15 KB)
